# Realizing luminescent single crystals of covalent organic polymers with color-tunable emission through isomer engineering

**DOI:** 10.1039/d5sc09875k

**Published:** 2026-03-03

**Authors:** Yanyan Qin, Pengfei She, Xiaoda Wang, Jianjun Wu, Mingbing Lian, Feiyang Li, Shujuan Liu, Qianfeng Gu, Yun Ma, Qiang Zhao, Qichun Zhang

**Affiliations:** a College of Electronic and Optical Engineering and College of Flexible Electronics (Future Technology), Nanjing University of Posts and Telecommunications Nanjing 210023 P. R. China pengfei.she@njupt.edu.cn iamyma@njupt.edu.cn iamqzhao@nuist.edu.cn; b Department of Materials Science and Engineering, City University of Hong Kong Tat Chee Avenue Kowloon Tong Hong Kong P. R. China qiczhang@cityu.edu.hk; c State Key Laboratory of Flexible Electronics (LoFE), Institute of Advanced Materials (IAM), Nanjing University of Posts and Telecommunications Nanjing 210023 P. R. China; d School of Environmental and Chemical Engineering, Jiangsu University of Science and Technology Zhenjiang 212100 P. R. China; e City University of Hong Kong Shenzhen Research Institute Shenzhen Guangdong Province 518057 P. R. China

## Abstract

Preparing luminescent single crystals of B–N-containing covalent organic polymers (COPs) is very important but highly challenging, since most conditions only produce luminescence-silent crystals. To address this issue, we employ an isomer engineering strategy to successfully prepare luminescent (CityU-50) and luminescence-silent (CityU-60) single crystals of B–N linked COPs based on two distinct chromophore isomers, 3,3′,6,6′-tetra(pyridin-4-yl)-9,9′-bicarbazole (3,6-TPy-BCz) and 2,2′,7,7′-tetra (pyridin-4-yl)-9,9′-bicarbazole (2,7-TPy-BCz). Single-crystal X-ray diffraction analysis reveals that 3,6-TPy-BCz can lead to a “loose” stacking model of helical polymer chains in CityU-50, effectively suppressing interchain aggregation and endowing CityU-50 with unprecedented visible-light-excitable luminescence, while 2,7-TPy-BCz results in a “close” packing model of helical polymer chains in CityU-60, leading to a non-emissive structure. This factor validates our design principle. We also find that CityU-50 can display excitation-dependent and solvent-responsive luminescence due to the different aggregation states within the crystal and solvent-triggered packing changes in the polymer chains of CityU-50. Based on these responses, we demonstrate the application of CityU-50 in advanced information encryption. This work not only delivers a new class of smart crystalline materials but also establishes a powerful strategy for engineering crystalline polymers with precisely tailored structures and properties.

## Abstract 2

Luminescent B–N linked polymer single crystals have been successfully synthesized through a molecular isomer engineering strategy. Moreover, advanced information encryption and optical storage applications have been developed.

## Graphical abstract

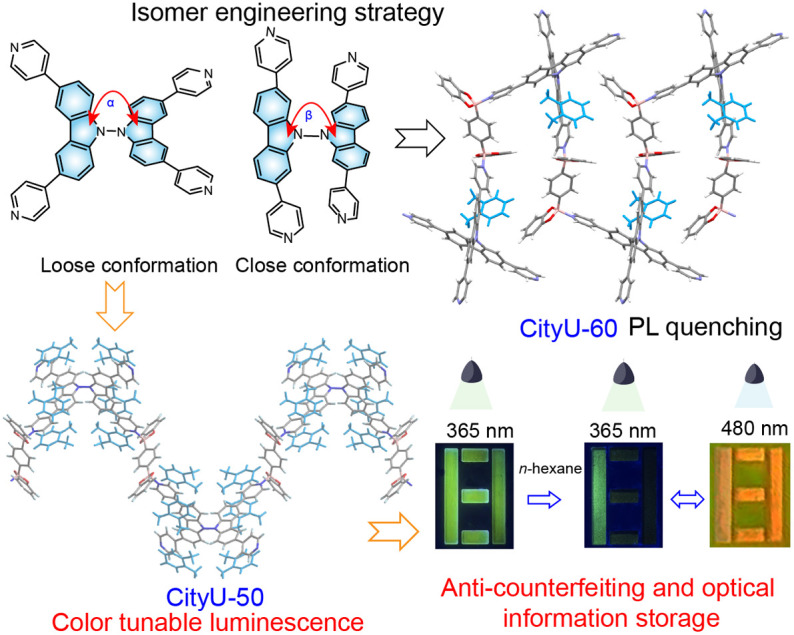

## Introduction

Photoluminescent (PL) organic single crystals have drawn great attention benefiting from their potential applications in cutting-edge technologies such as optical waveguides, optical communication, displays and advanced information encryption.^1–5^ However, it is a challenge to obtain luminescent single crystals of covalent organic polymers/frameworks (COPs/COFs) because of the aggregation-caused quenching (ACQ) effect, a common phenomenon in conjugated polymers, and the inherent synthetic difficulties in growing long-range crystalline order with desired functionalities.^6–8^ To elucidate the underlying PL quenching mechanism, growing luminescent single crystals of COPs for single-crystal X-ray diffraction (SCXRD) analysis is extremely necessary because this technique enables the accurate identification of geometric factors such as atomic positions, bond lengths and angles, stacking patterns and crucial host-guest molecular interactions.^9–12^ Importantly, the detailed structural information not only helps to understand the growing kinetics and thermodynamics of single crystals but also offers a well-established platform to elucidate the relationships among building blocks, structures, and luminescent properties.

**Fig. 1:**
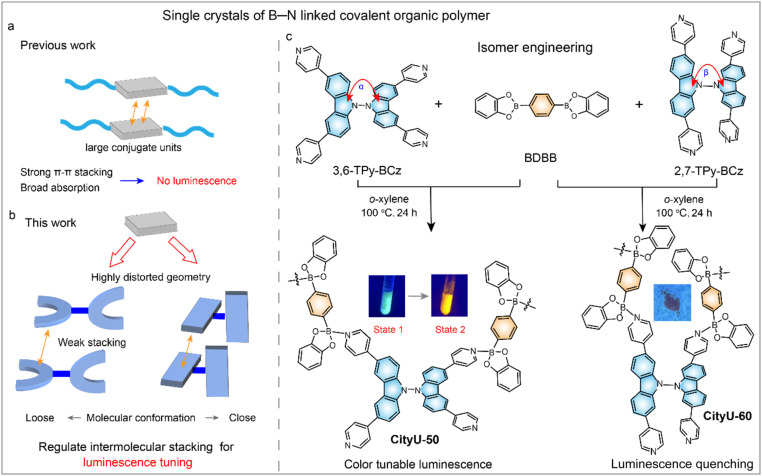
Schematic illustration of the design strategy for luminescent COPs. (a and b) Schematic design of distorted building units with loose and close conformations, regulating intermolecular stacking of COPs for luminescence tuning. (c) Synthetic routes to CityU-50 and CityU-60. The left insets are photographs of CityU-50 before and after treatment with *n*-hexane under UV and visible light. State 1 refers to CityU-50 crystals without any treatment, while State 2 refers to CityU-50 crystals that were soaked in *n*-hexane at room temperature for 1 h. The right inset is a photograph of CityU-60 under UV or visible light.

Currently, highly reversible covalent bonds, such as imine linkages, boron ester linkages and dative B–N bonds, have been extensively utilized to grow single crystals of COPs by facilitating efficient self-correction of defects during the self-assembly process.^13–20^ Compared to imine (∼600 kJ mol^−1^) and boronate ester (∼500 kJ mol^−1^) bonds, dative B–N bonds possess relatively low binding energy (approximately 100 kJ mol^−1^). This lower energy facilitates the formation of large single crystals through reversible self-assembly. Although a series of large single crystals of functional COPs featuring B–N bonds have been extensively reported, the luminescence properties remain scarcely mentioned (Fig. 1a).^21–32^ Besides, developing stimuli-responsive luminescent COPs that are capable of dynamically modulating their emission properties in response to external stimuli is particularly interesting for next-generation photonic applications, notably in anti-counterfeiting technologies.^33,34^

Here, we address this long-standing issue by proposing an isomer engineering strategy. We designed two highly distorted chromophore isomers, a “loose” 3,3′,6,6′-tetra(pyridin-4-yl)-9,9′-bicarbazole (3,6-TPy-BCz) and a “close” 2,2′,7,7′-tetra(pyridin-4-yl)-9,9′-bicarbazole (2,7-TPy-BCz). We hypothesized that the sterically bulky, loose conformation of 3,6-TPy-BCz would effectively disrupt intermolecular π-π stacking to overcome ACQ, while simultaneously creating sufficient free volume to accommodate external stimuli (Fig. 1b). Then, two single crystals of B–N linked COPs (luminescent CityU-50 and non-emissive CityU-60) were prepared using chromophore isomers and 1,4-bis(benzo[*d*][1,2,3]dioxaborol-2-yl)benzene (BDBB) as building units (Fig. 1c). As expected, CityU-50 exhibits significant visible-light-excitable green emission, while CityU-60 is completely non-emissive. SCXRD analysis reveals that the persistence of a solvent sheath of six *o*-xylene molecules encapsulating each unit in CityU-50 effectively isolates the polymer chains and impedes detrimental interchain π–π stacking. In contrast, in its “close” isomer-based counterpart (CityU-60), many π–π interactions exist between adjacent polymer chains, perfectly validating our design principle. Interestingly, CityU-50 displays excitation-dependent and solvent-responsive luminescence behaviors. Based on the feature of color-tunable luminescence in CityU-50, we demonstrate its application in advanced information encryption. This work not only delivers a new class of functional crystalline materials but also establishes a powerful design strategy for engineering smart luminescent polymers with precisely tailored structures and properties.

## Results and discussion

The single crystals of CityU-50 and CityU-60 were firstly prepared using distinct chromophore isomers (3,6-TPy-BCz and 2,7-TPy-BCz) and BDBB as building blocks under solvothermal conditions. Briefly, 3,6-TPy-BCz or 2,7-TPy-BCz and BDBB (molar ratio = 1 : 1) were mixed in dry *o*-xylene and heated at 100 °C for 24 hours. After cooling to room temperature, large colorless single crystals (up to 5 mm) of CityU-50 were formed at the bottom of the reaction vessel, whereas small faint yellow single crystals (up to 3 mm) of CityU-60 were found. The synthesis procedure is illustrated in Fig. 1c. The detailed experimental process as well as the characterization data are provided in the SI (Scheme S1 and Fig. S1–S6).

**Fig. 2:**
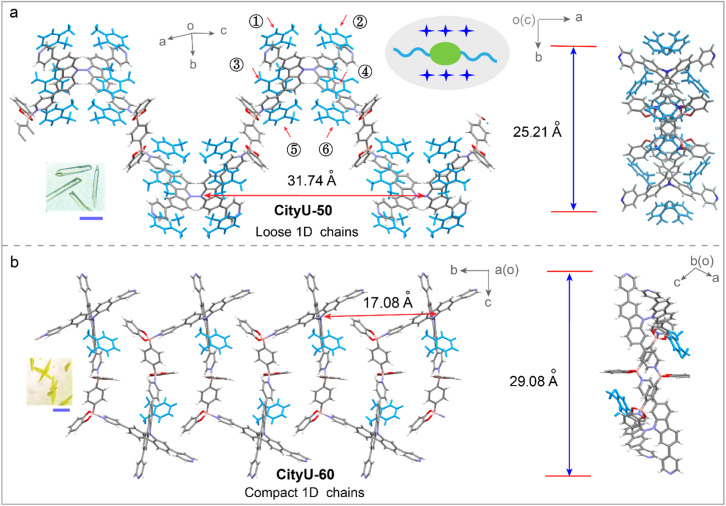
The 1D chains of CityU-50 (a) and CityU-60 (b). Inset: photos of single crystals of CityU-50 and CityU-60. Scale bar = 3 mm.

To elucidate the impact of isomer engineering on structural characteristics, SCXRD analysis of CityU-50 and CityU-60 was conducted. The data analysis reveals that CityU-50 crystallizes in the monoclinic *C*2/*c* space group with lattice constants of *a* = 24.5308(4) Å, *b* = 10.3508(2) Å, and *c* = 31.7355(6) Å, and a unit-cell volume of 7507.2(3) Å^3^ (Table S1). The asymmetric unit of CityU-50 consists of one 3,6-TPy-BCz moiety, two half BDBB species, and two *o*-xylene molecules (Fig. S7a). Notably, within the 3,6-TPy-BCz building block, four pyridine rings exhibit a significantly twisted arrangement, characterized by dihedral angles of 27.50 and 10.98° (Fig. S7b). The dihedral angles of two carbazole planes and bond lengths of N–N between two carbazole planes are 89.43° and 1.38 Å, respectively. Further analysis reveals that one 3,6-TPy-BCz unit connects two BDBB species *via* dative B–N bonds with a B–N bond length of 1.66 Å, forming 1D helical chains with a helical pitch of 25.21 Å along the *c*-axis (Fig. 2a). This helical architecture, arising from the twisted 3,6-TPy-BCz units and B–N linkages, inherently creates an internal free volume within the COP chains. This helical architecture enforces a remarkable separation between the chromophores, with the distance between adjacent 3,6-TPy-BCz units along the *c*-axis reaching 31.74 Å. Importantly, this separation is maintained by a solvent sheath of six *o*-xylene molecules encapsulating each unit, which could contribute to isolating the polymer chains and impeding detrimental interchain π–π stacking. On the other hand, SCXRD measurement reveals that CityU-60 adopts a monoclinic *P*2_1_/*n* space group with lattice constants of *a* = 16.4184(2) Å, *b* = 17.0819(2) Å, and *c* = 21.1989(2) Å, and a unit-cell volume of 5687.75(11) Å^3^ (Table S2). The asymmetric unit of CityU-60 consists of one 2,7-TPy-BCz moiety, one BDBB species, and one *o*-xylene molecule. Unlike CityU-50, CityU-60 exhibits a markedly smaller dihedral angle of 74.73° between its two carbazole planes (Fig. S8), indicating that its more compact geometry is predicted to favor aggregation. Indeed, SCXRD analysis of CityU-60 reveals a dramatically different packing motif. Instead of isolated helical chains, CityU-60 forms compact, 1D wave-like chains (Fig. 2b). This conformation drastically reduces the inter-chromophore distance along the *c*-axis to 17.08 Å, nearly half that of CityU-50.

**Fig. 3:**
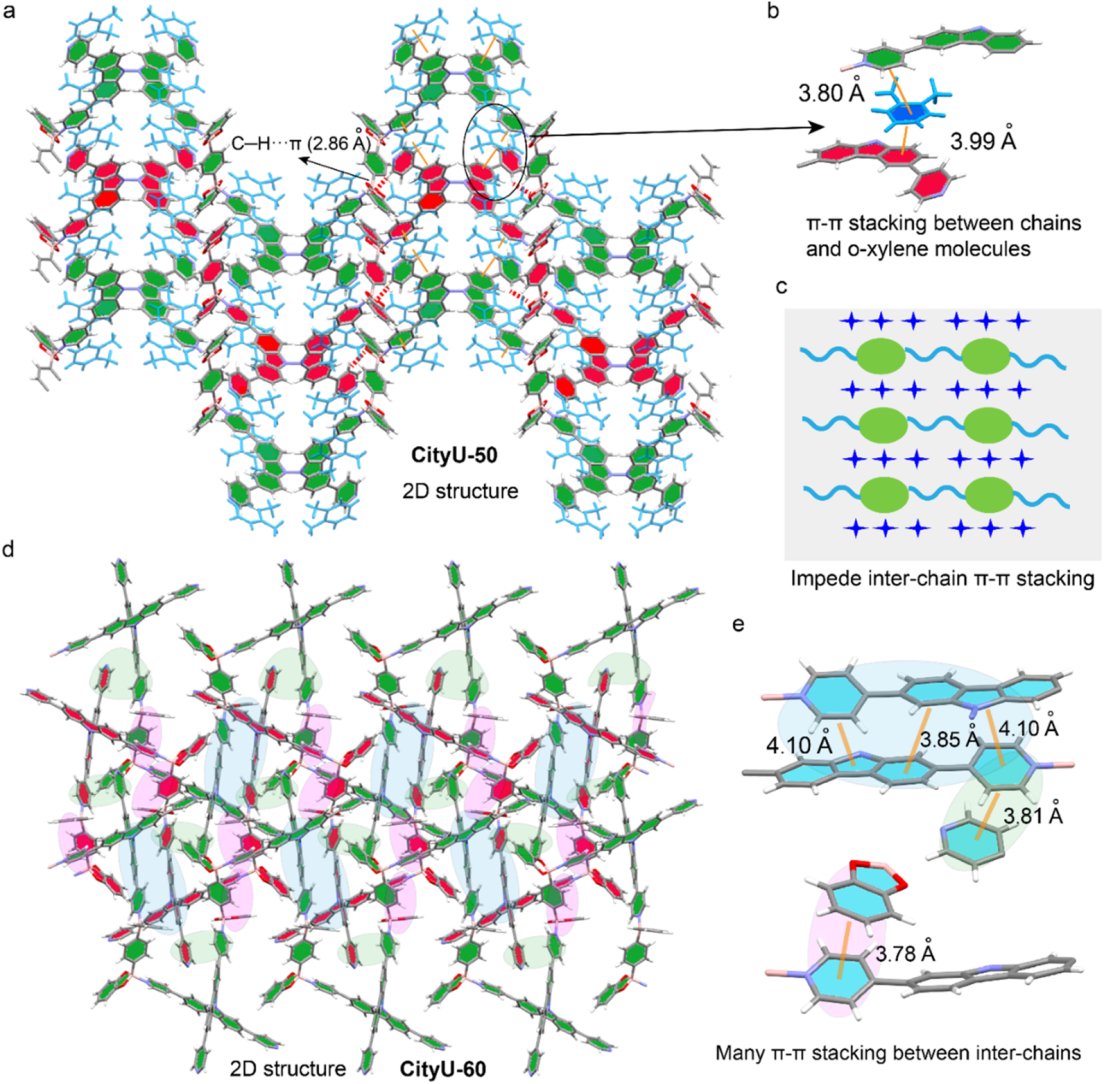
The 2D structures and stacking modes of CityU-50 (a–c) and CityU-60 (d and e).

Furthermore, the space arrangements of CityU-50 and CityU-60 also exhibit a significant difference. For CityU-50, the coordinated pyridine rings and carbazole planes within a single chain allow the formation of tight π–π interactions with adjacent *o*-xylene rings with short distances of 3.99 Å and 3.80 Å, respectively (Fig. 3a and b). These π–π interactions, mediated by *o*-xylene molecules, can bridge adjacent helical chains, contributing to the formation of a pseudo-2D layer. In addition, the C–H⋯π interactions between the benzene rings from BDBB within a single chain and free pyridine molecules in adjacent chains further stabilize this pseudo-2D layer. The presence of solvent molecules within the crystal lattice also suggests potential for solvent-responsive behavior. Moreover, the pseudo-2D layers are interconnected through C–H⋯O interactions between the oxygen atoms of B-aryl rings and *o*-xylene molecules, as well as C–H⋯C interactions between adjacent *o*-xylene molecules, resulting in the formation of a supramolecular architecture (Fig. S9). Remarkably, no π–π interaction between adjacent polymer chains is found in the crystals of CityU-50 (Fig. 3c). Unlike the solvent-mediated assembly in CityU-50, the pseudo-2D layers in CityU-60 are formed through extensive, direct π–π interactions (3.78–4.10 Å) among BDBB, pyridine rings and carbazole planes in adjacent polymer chains (Fig. 3d and e). Furthermore, independent gradient model (IGM) analysis confirms this stark difference, visualizing widespread π–π interactions between pyridine and carbazole moieties in CityU-60, which are conspicuously absent in CityU-50 (Fig. S10). Besides, the experimental powder X-ray diffraction (PXRD) results and the simulated patterns match each other well, indicating that the as-synthesized CityU-50 and CityU-60 samples exhibit high phase purity (Fig. S11 and S12). Field-emission scanning electron microscopy (FESEM) analysis reveals that the CityU-50 crystals possess a cubic-like 3D morphology (Fig. S13). Energy dispersive spectroscopy (EDS) mapping confirms the existence of oxygen, nitrogen and boron elements in the B–N crystal. Besides, the porosities of CityU-50 and CityU-60 single crystals were further investigated *via* a nitrogen adsorption experiment at 77 K. The isotherms and pore size distribution curves, calculated by the Brunauer–Emmett–Teller (BET) and Barrett–Joyner–Halenda (BJH) methods, are presented in Fig. S14. CityU-50 possesses a small BET surface area of 4.51 m^2^ g^−1^, along with a pore volume of 0.0092 cm^3^ g^−1^, while the CityU-60 exhibits a BET surface area of 7.13 m^2^ g^−1^ and pore volume of 0.016 cm^3^ g^−1^, suggesting that both polymers are virtually nonporous. Thus, we believe that this profound structural discrepancy between CityU-50 and CityU-60 should directly affect their optical properties due to a possible ACQ effect.

**Fig. 4:**
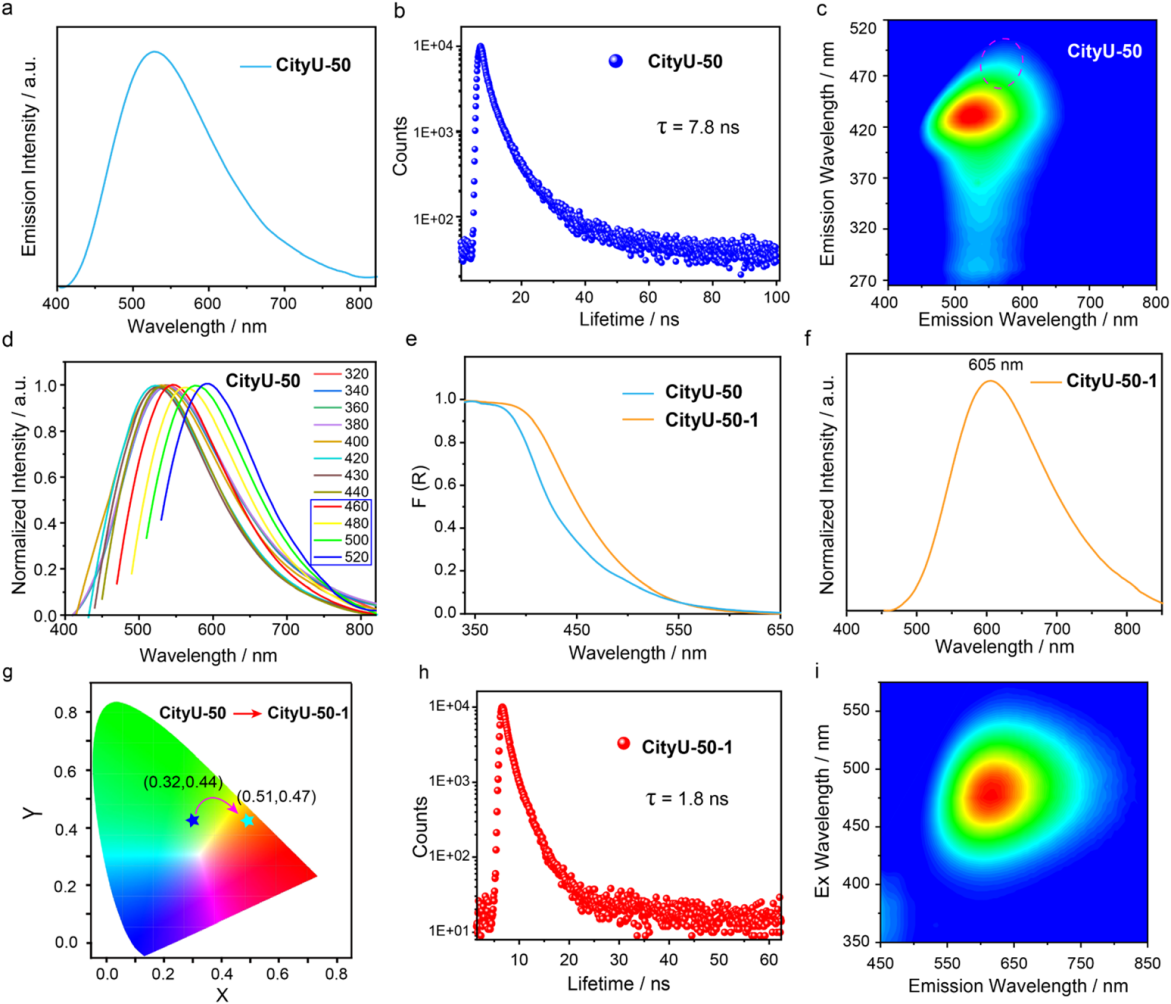
The photoluminescent properties of CityU-50 and CityU-50-1 (CityU-50 after treatment with *n*-hexane). (a) Photoluminescence, (b) lifetime decay profiles (535 nm), (c) mapping of excitation-photoluminescence and (d) excitation-dependent photoluminescence of CityU-50, recorded under ambient conditions at 298 K. (e) UV-Vis diffuse reflectance spectra of CityU-50 and CityU-50-1 converted using the Kubelka–Munk function, F(R). (g) CIE coordinate diagram of CityU-50 and CityU-50-1. (f) Photoluminescence, (h) lifetime decay profiles (605 nm) and (i) mapping of excitation-photoluminescence of CityU-50-1, recorded under ambient conditions at 298 K.

To validate our hypothesis, the photoluminescence (PL) properties of CityU-50 and CityU-60 samples were investigated. Excitingly, CityU-50 exhibits intense green emission under 365 nm UV irradiation, with the primary emission peak at ∼535 nm (Fig. 4a). Meanwhile, the 3,6-TPy-BCz monomer displays a significantly blue-shifted emission peak at 385 nm, thereby excluding the possibility that the green emission originates from the 3,6-TPy-BCz monomer (Fig. S15). The photoluminescence quantum yield (PLQY) and lifetime at 535 nm are measured to be 4.43% and 7.8 ns (Fig. 4b), respectively, confirming its fluorescence feature. From the excitation-luminescence mapping of CityU-50 (Fig. 4c), it is evident that the optimized excitation wavelength is 435 nm, indicating that the green emission from CityU-50 can be efficiently triggered by a visible light source. In contrast, a complete shutdown of optical emission is observed in CityU-60 sample (Fig. S16), indicating that suppressing interchain stacking is key to luminescence. Meanwhile, this stark, “lights-off” result in CityU-60, when contrasted with the bright, tunable emission of CityU-50, provides unambiguous evidence that the isomer molecular conformation of the building block directly governs the packing of polymers, which in turn dictates its luminescent properties. Interestingly, it is found that the CityU-50 sample exhibits the obviously color tunable luminescence feature upon multiple external stimuli. As shown in Fig. 4d and S17, different from excitation wavelengths ranging from 320 to 440 nm, CityU-50 exhibits excitation-dependent PL behavior within the range of 440–520 nm. Specifically, CityU-50 displays an orange emission peak at 595 nm when excited at 520 nm. This excitation-dependent luminescence may likely originate from distinct emissive species associated with different aggregation states within the crystal,^35–37^ generated by crystal defects stemming from the loss of *o*-xylene. Therefore, the dominant green emission of the CityU-50 sample may also be fully modified to dominant orange emission by the exchange or removal of *o*-xylene. To confirm this guess, CityU-50 crystals were soaked in *n*-hexane at room temperature for 1 h, and the corresponding solid-state absorption and PL spectra were recorded. The sample treated with *n*-hexane is referred to as CityU-50-1. As depicted in Fig. 4e, CityU-50-1 exhibits a distinct redshift in its absorption spectrum compared to CityU-50, suggesting a potential alteration in the electronic transitions resulting from changes in either molecular structure or intermolecular stacking arrangements. Importantly, a notable redshift in the luminescence spectra is observed, with the main emission peak shifting from 535 nm to 605 nm (Fig. 4f). This spectral shift is accompanied by a change in emission color from green to orange, with the corresponding CIE coordinates shifting from (0.32, 0.44) to (0.51, 0.47) (Fig. 4g). Furthermore, the PLQY and lifetime at 605 nm of CityU-50-1 are measured to be 5.58% and 1.8 ns, respectively (Fig. 4h). Unlike CityU-50, the luminescence of CityU-50-1 can only be excited by visible light, and no obvious excitation-dependent luminescence behavior is observed (Fig. 4i and S18). This suggests that the solvent-induced structural change in CityU-50-1 leads to a more homogeneous aggregation state, resulting in a single dominant emissive species and the elimination of excitation-dependent luminescence. To elucidate whether the exchange or removal of *o*-xylene in CityU-50 induces this color-tunable luminescence, a series of control experiment were carried out. Firstly, the luminescence behavior of CityU-50 was monitored in different solvents. It's found that smaller solvent molecules more readily induce the luminescence color response (Fig. S19). Subsequently, Fourier-transform infrared spectra (FTIR) of CityU-50 and CityU-60 exhibit minimal variation, indicating that the chain structure of CityU-50 remains essentially intact after *n*-hexane exposure (Fig. S20). Time-dependent PXRD measurements on CityU-50 soaked in *n*-hexane for different durations were carried out. As shown in Fig. S21, with increasing soaking time, broad bands gradually appear in the range of 15–30°. Moreover, the original diffraction peaks remain discernible even after prolonged *n*-hexane exposure. These results indicate that prolonged exposure to *n*-hexane leads to the gradual collapse of the molecular stacking of CityU-50. Furthermore, we attempted to directly remove *o*-xylene solvent molecules from single crystals of CityU-50 by heating and subsequently investigate the photophysical properties. After heating single crystals of CityU-50 at 150 °C for 1 h, we found that the PXRD pattern, absorption, and luminescence of the sample are similar to those observed after *n*-hexane treatment (CityU-50-1), rather than those of the as-synthesized CityU-50 (Fig. S22 and S23). Furthermore, we conducted time-dependent density functional theory (TDDFT) calculations on monomer and dimer fragments. The results show that dimer fragments exhibit a lower energy level in the excited singlet state relative to the monomer (Fig. S24). Thus, the above-mentioned results clearly demonstrate that the unique solvent-responsive luminescence behavior of CityU-50 can be attributed to the aggregation of polymer chains within the crystal structure, triggered by the exchange and removal of *o*-xylene molecules, which induces a change in intermolecular packing and enhances intermolecular interactions. Meanwhile, the excitation-dependent luminescence behavior of CityU-50 should be attributed to the presence of multiple aggregation states within the crystal lattice. These distinct aggregation states, potentially varying in their extent and influenced by factors such as crystal defects resulting from the partial loss of *o*-xylene molecules, could give rise to different emissive species, thus accounting for the observed excitation-dependent emission.

**Fig. 5:**
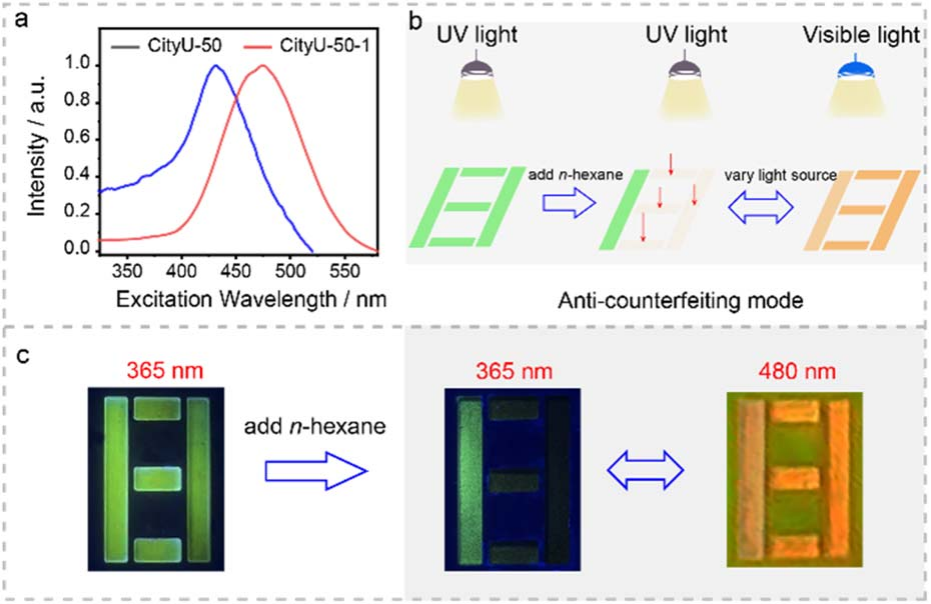
Optical application. (a) Excitation spectra of CityU-50 and CityU-50-1 (CityU-50 after treatment with *n*-hexane). (b and c) Visual representation illustrating the process of information encryption and decryption.

Given the distinctive color tunable luminescence behaviors of CityU-50, we exploited its promising applications in anti-counterfeiting and optical information storage (Fig. 5a). As illustrated in Fig. 5b, the encryption digit “8” was prepared using CityU-50 microcrystals. Initially, under 365 nm UV light illumination, the digit “8” exhibits a pronounced green emission. Subsequently, upon adding *n*-hexane to a part of the pattern, only the digit “1” remains visible (Fig. 5c). However, under 480 nm visible light illumination, the digit “8” with orange emission becomes discernible. This result highlights the outstanding potential of the tunable luminescent B–N polymer single crystals for advanced optical information storage and encryption applications.

## Conclusions

In conclusion, by introducing two distorted chromophore isomers with different conformations as building units, we have demonstrated that loose and close conformations have a dramatic impact on the single-crystal structures and optical properties of COPs. SCXRD analysis shows that CityU-50 with a loose conformation is a 1D helical polymer, where *o*-xylene solvent molecules play a critical role in directing the crystal packing and supramolecular architecture, while the dense packing and pervasive π–π interactions between adjacent 1D chains are found in the pseudo-2D layer of CityU-60 with a close conformation. Consequently, CityU-50 exhibits excitation-dependent and solvent-responsive luminescence. Comprehensive experimental and theoretical investigations indicate that the excitation-dependent luminescence arises from the presence of multiple aggregation states within the crystal lattice, and solvent-responsive luminescence is attributed to an aggregation of polymer chains, triggered by the exchange of lattice-confined *o*-xylene molecules. Based on this distinctive tunable luminescence, we have demonstrated its application in advanced information encryption and optical storage areas. Significantly, this work not only showcases the practical utility of CityU-50 but also establishes a robust design principle for engineering stimulus-responsive luminescent crystalline polymers based on distorted chromophores and dynamic B–N linkages.

## Author contributions

P. She, Y. Ma, Q. Zhao and Q. Zhang conceived the project and experiments. Y. Qin designed the molecules. Y. Qin and X. Wang conducted the experiment and wrote the manuscript. S. Liu helped revise the manuscript. J. Wu, M. Lian and Q. Gu performed the optical application. F. Li conducted the theoretical calculation. All authors contributed to writing the manuscript.

## Conflicts of interest

There are no conflicts to declare.

## Supplementary Material

SC-OLF-D5SC09875K-s001

SC-OLF-D5SC09875K-s002

## Data Availability

CCDC 2414993 and 2465520 contain the supplementary crystallographic data for this paper.^38*a*,*b*^ The authors confirm that the data supporting the findings of this study are available within the article and its supplementary information (SI). Supplementary information: synthetic procedures, experimental details and supplemental figures. See DOI: https://doi.org/10.1039/d5sc09875k.
