# Supplementary material for: Realizing luminescent single crystals of covalent organic polymers with color-tunable emission through isomer engineering
Source: Chem Sci. 2026 Mar 3;17(17):8662–9. doi: 10.1039/d5sc09875k (PMC12973223; doi:10.1039/d5sc09875k)
Supplement: SC-017-D5SC09875K-s001 [file SC-017-D5SC09875K-s001.pdf]

Supplementary Information (SI) for Chemical Science.
This journal is © The Royal Society of Chemistry 2026

Supporting Information

Realizing luminescent single crystals of covalent organic polymers with color-tunable emission through isomer engineering

Yanyan Qin,[abc] Pengfei She,[*abc] Xiaoda Wang,[ac] Jianjun Wu,[ac] mingbing Lian,[ac] Feiyang Li,[d] Shujuan Liu,[c] Qianfeng Gu,[b] Yun Ma,[*ac] Qiang Zhao[*ac] and Qichun Zhang*[be]

[a]*College of Electronic and Optical Engineering and College of Flexible Electronics (Future Technology), Nanjing University of Posts and Telecommunications, Nanjing 210023, P. R. China.*

[b]*Department of Materials Science and Engineering, City University of Hong Kong, Tat Chee Avenue, Kowloon Tong, Hong Kong P. R. China*

[c]*State Key Laboratory of Flexible Electronics (LoFE) & Institute of Advanced Materials (IAM), Nanjing University of Posts and Telecommunications, Nanjing 210023, P. R. China.*

[d]*School of Environmental and Chemical Engineering, Jiangsu University of Science and Technology, Zhenjiang 212100, P. R. China.*

[e]*City University of Hong Kong Shenzhen Research Institute Shenzhen, Guangdong Province, 518057, P. R. China.*

1. Experimental Procedures

1.1. Materials and reagents

All commercially available chemicals and reagents were used without further purification. Dry o-xylene was purchased from Sigma Aldrich and used as received.

1.2. Instrumentation and characterizations

All characterization measurements for monomers in this study were performed at room temperature (298 K). ^{1}H and ^{13}C NMR spectra were recorded with Bruker 400 MHz AVANCE III Nuclear Magnetic Resonance System. Fourier transform infrared (FT-IR) spectra were recorded on an Agilent-Cary 670 FT-IR spectrometer in transmission mode, and the conventional KBr plate method. Elemental analysis was measured by an Agilent 710 Series Elemental Analyzer. Powder X-ray diffraction (PXRD) measurement was recorded on a Rigaku Smartlab 9 kW X-ray diffractometer using CuKα radiation (λ = 0.154 nm) at 40 kV and 30 mA at RT. Thermogravimetric analysis (TGA) was performed using a TAQ-50 analyzer under a flow of N_2 at a heating rate of 5 °C min^{-1}. UV–vis spectra were recorded on a Shimadzu UV-2600 spectrophotometer from reflection solid-state measurements. Steady-state photoluminescence (PL) emission spectra, PL decay spectra and quantum yield measurements were recorded using Edinburgh FLS1000 spectrophotometer.

1.3. Single-Crystal X-ray Analyses. The diffraction data of **CityU-50** single crystal were carried out at 150 K with a Bruker D8 Venture diffractometer, equipped with a PHOTON III detector using the monochromatized wavelength (Cu Kα) = 1.54178 Å. The crystal structures were solved and refined by full matrix least-squares methods against F2 using the SHELXL-2018 program package16 and Olex-2 software. All non-hydrogen atoms were refined with anisotropic displacement parameters and hydrogen positions were fixed at calculated positions and refined. The crystallographic data and structure refinement for the polymer is summarized in Table S1.

1.4. Calculations details. All theoretical calculations were carried out using the Gaussian 09 software package. The B3LYP functional was used in the density functional theory (DFT) calculations with the D3(BJ) dispersion correction. The def2-SVP basis set was utilized in geometry optimizations and frequency analyses for all atoms. Vertical excitation energies were computed via time-dependent DFT (TD-DFT) with the M062X functional. Further, based on dimer fragments from the single crystal of COPs without any optimization, analysis and visualization of the independent gradient model (IGM) were finished by Multiwfn 3.7(dev) and VMD based.

1.5. Preparation of monomers and polymer crystal

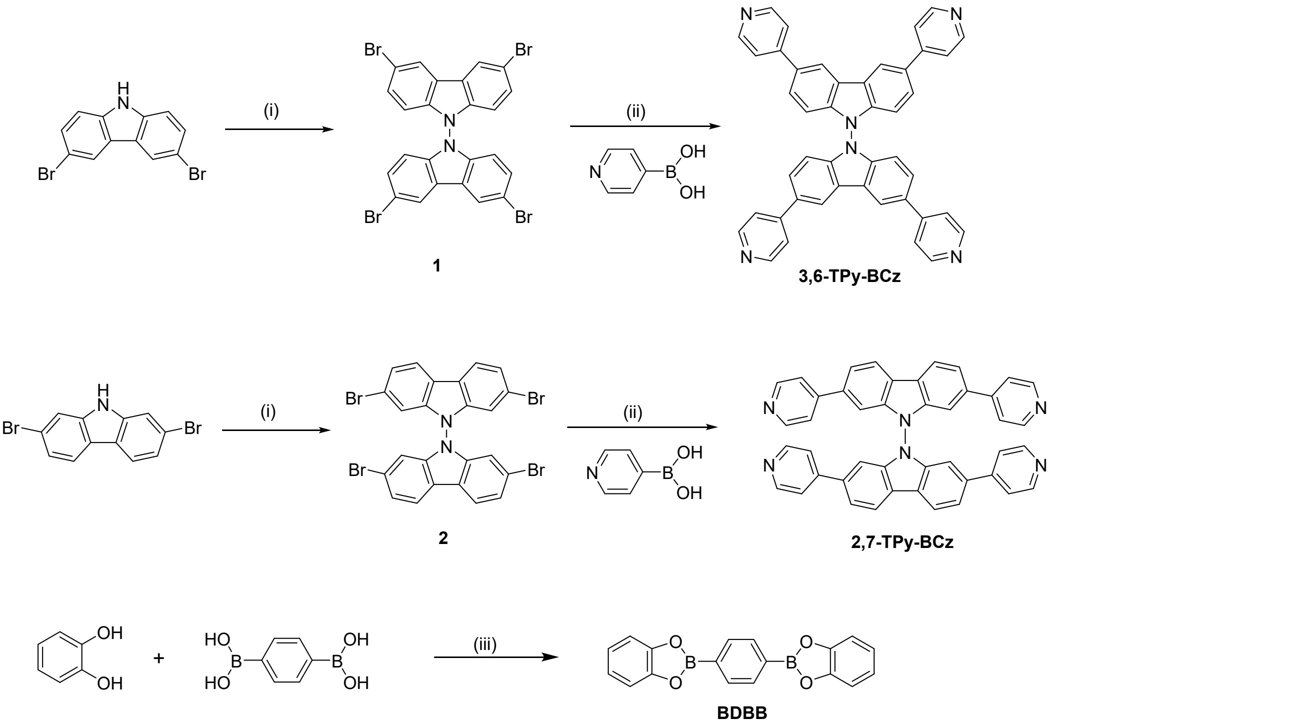

Scheme S1. Synthetic routes for two monomers 3,6-TPy-BCz and 2,7-TPy-BCz; (i) $KMnO_4$, acetone, 80 °C, 5 h; (ii) K_2CO_3, $PdCl_2$(dppf), THF/H_2O, 60 °C; (iii) toluene, 110 °C, 24 h.

Synthesis of compound 1. Potassium permanganate (2.37 g, 15 mmol) was added to a solution of 3,6-dibromocarbazole (1.63 g, 5 mmol) in 25 ml acetone and then stirred for 5 h at 80 ^{o}C. The reaction mixture was cooled to 25 °C and the solvent was evaporated under vacuum. The residue was further extracted with $CHCl_3$ for 12 h with stirring, then washed three times with $CHCl_3$. The solvent was removed and the residue was purified by silica gel column chromatography. The off-white product of compound **1** was obtained after drying; yield: 0.97 g (60%). ^{1}H NMR (400 MHz, $CDCl_3$): δ = 8.27 (s, *J* = 1.4 Hz, 4 H), 7.47 (dd, *J* = 1.9 Hz, 8 Hz, 4 H), 6.75 (d, *J* = 8 Hz, 4 H). ^{13}C NMR (100 MHz, $CDCl_3$): δ = 138.63, 130.53, 124.08, 122.80, 115.13, 110.67.

Synthesis of monomer 3,6-TPy-BCz. In N_2 atmosphere, compound **1** (3.24 g, 5 mmol) was dissolved in a THF/H_2O (v/v, 5:1, 30 mL). Then, pyridin-4-ylboronic acid (2.58 g, 21 mmol), K_2CO_3 (2.90 g, 21 mmol), and $PdCl_2$(dppf) (180 mg, 0.25 mmol) were added. The reaction mixture was refluxed at 60 °C for 72 h and monitored by thin-layer chromatography. After cooling to 25 °C, H_2O was added into the solution, the crude product was separated through filtration, and treated with a small amount of MeOH; the mixture was heated until it boiled, and then the solution was sonicated for 15 min. The precipitate was collected by filtration, and further washed with H_2O, ethanol and acetone, respectively, then vacuum dried, giving a white solid, yield: 2.53 g (79%). ^{1}H NMR (400 MHz, $CDCl_3$): δ = 8.72 (d, *J* = 8 Hz, 8 H), 8.58 (s, 4 H), 7.71 (d, *J* = 8 Hz, 4 H), 7.65 (d, *J* = 8 Hz, 8 H), 7.10 (d, *J* = 8 Hz, 4 H). ^{13}C NMR (100 MHz, $CDCl_3$): δ = 150.54, 148.55, 140.66, 132.74, 126.69, 122.87, 121.96, 119.91, 109.97.

Synthesis of compound 2. This compound was prepared similarly as compound **1**, giving an off-white solid with yield: 0.78 g (45%). ^{1}H NMR (400 MHz, $CDCl_3$): δ = 8.03 (d, *J* = 8 Hz, 4 H), 7.51 (dd, *J* = 4 Hz, 8 Hz, 4 H), 7.01 (d, *J* = 1.6 Hz, 4 H).

Synthesis of monomer 2,7-TPy-BCz. This compound was prepared similarly as **3,6-TPy-BCz**, giving an off-white solid with yield: 1.76 g (55 %). ^{1}H NMR (400 MHz, $CDCl_3$): δ = 8.55 (d, *J* = 8 Hz, 8 H), 8.39 (d, *J* = 8 Hz, 4 H), 7.73 (dd, *J* = 4 Hz, 8 Hz, 4 H),

7.41 (d, J = 4 Hz, 8 H), 7.19 (s, 4 H). ^{13}C NMR (100 MHz, $CDCl_3$): δ = 149.19, 146.89, 139.75, 136.82, 121.21, 120.97, 120.85, 120.32, 106.08.

Synthesis of 1,4-bis(benzo[d][1,3,2]dioxaborol-2-yl)benzene (BDBB). A mixture of 1,4-phenylenebisboronic acid (3.32 g, 20 mmol) and catechol (2.02 g, 20 mmol) were dissolved in 120 mL toluene, and the mixture was heated at 110 °C with a Dean-Stark apparatus device for 48 h. After cooled to RT, the solid was collected by filtration, then vacuum dried, giving a grey solid, yield: 5.21 g (83%). ^{1}H NMR (400 MHz, $CDCl_3$) δ = 8.23 (s, 4H), 7.39-7.34 (m, 4H), 7.21-7.15 (m, 4H).

Preparation of B-N polymer crystals. The single crystals of **CityU-50** and **CityU-60** were prepared through a solvothermal method. In detail, a mixture of 3,6-TPy-BCz or 2,7-TPy-BCz (5 mg, 7.8 mmol) and BDBB (2.46 g, 7.8 mmol) were dissolved in 2 mL o-xylene, and the mixture was heated at 100 °C for 24 h. After cooling to room temperature, large colorless single crystals (up to 5 mm) of **CityU-50** were formed at the bottom of the reaction vessel, whereas small faint yellow single crystals (up to 3 mm) of **CityU-60** were found. These crystal were collected by sucking filtration and thoroughly washed with o-xylene, then vacuum dried. Yield: 5.7 mg (77%) and 5.2 mg (69%).

2. Characterizations

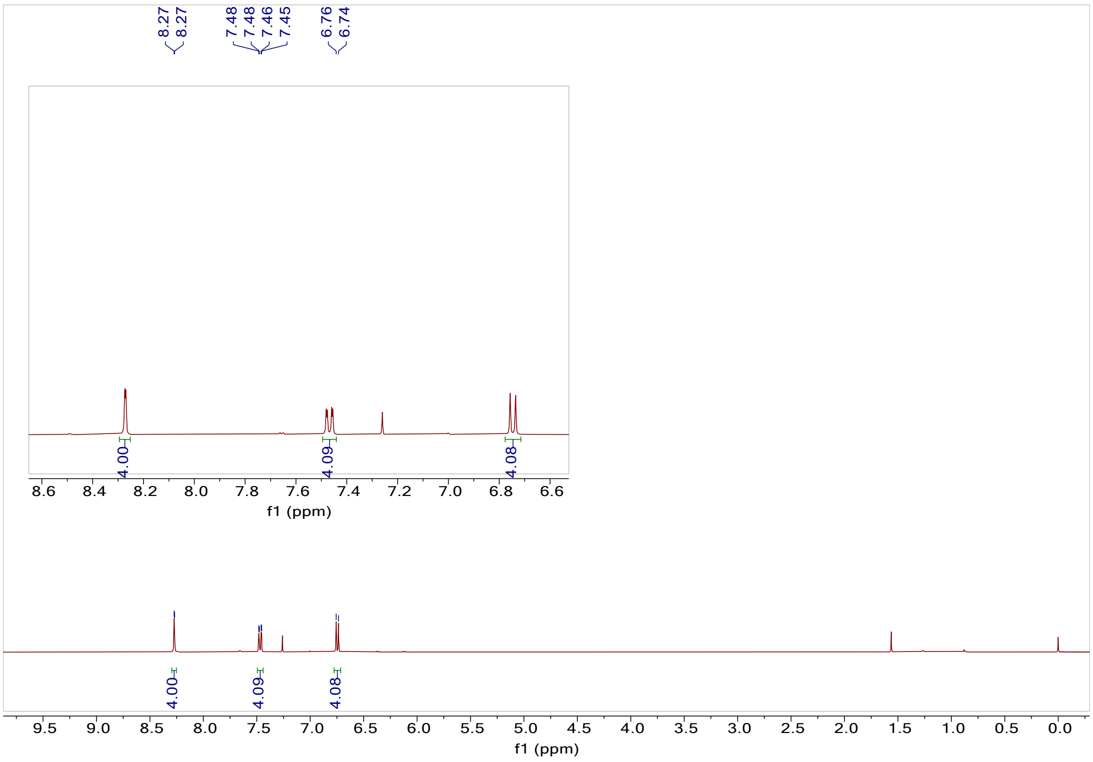

Fig. S1. ^{1}H NMR (400 MHz) spectrum of compound **1** recorded in $CDCl_3$ at RT.

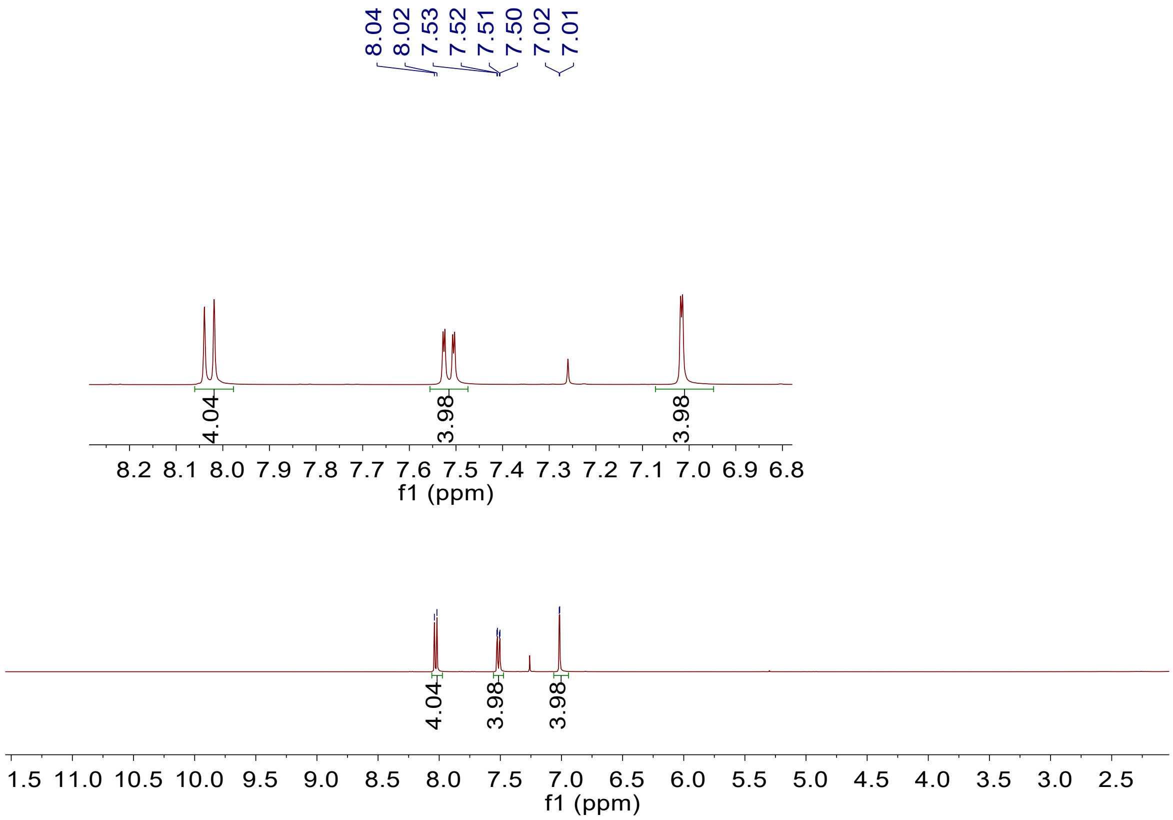

Fig. S2. ^{1}H NMR (400 MHz) spectrum of compound **2** recorded in $CDCl_3$ at RT.

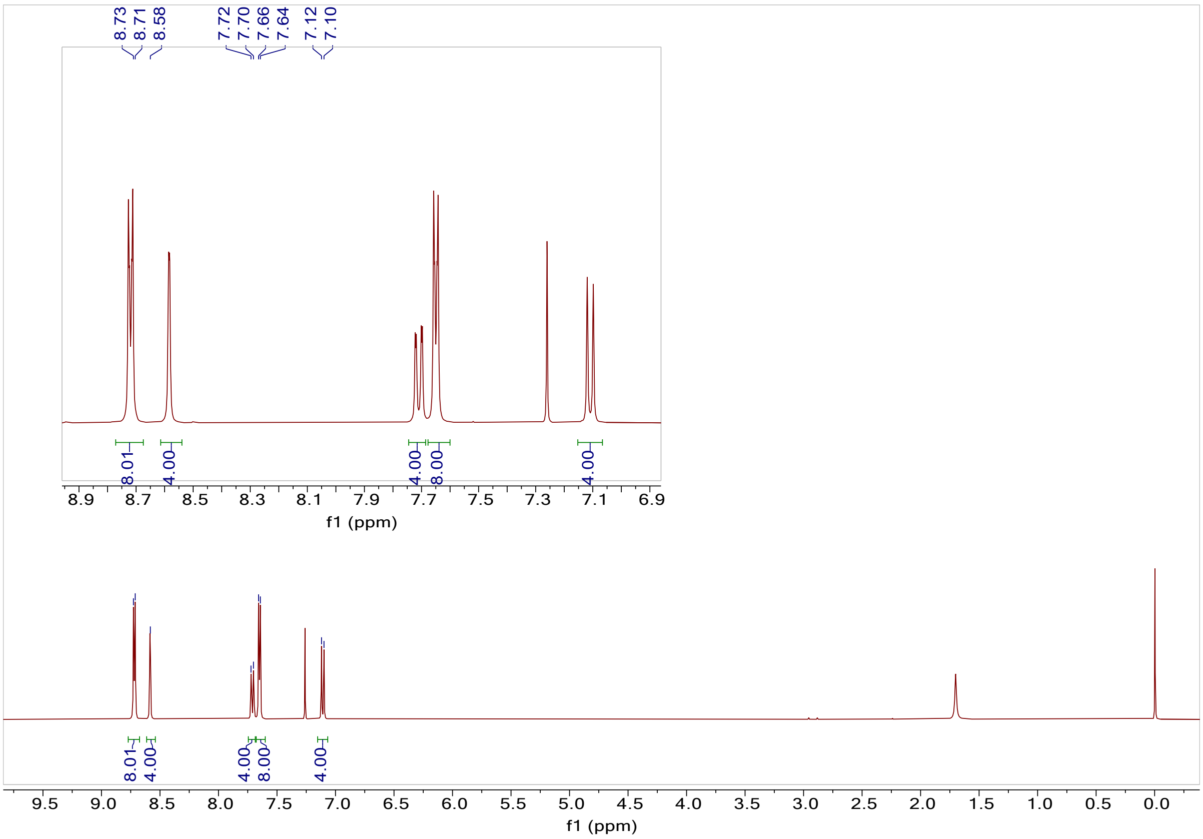

Fig. S3. ^{1}H NMR (400 MHz) spectrum of monomer **3,6-TPy-BCz** recorded in $CDCl_3$ at RT.

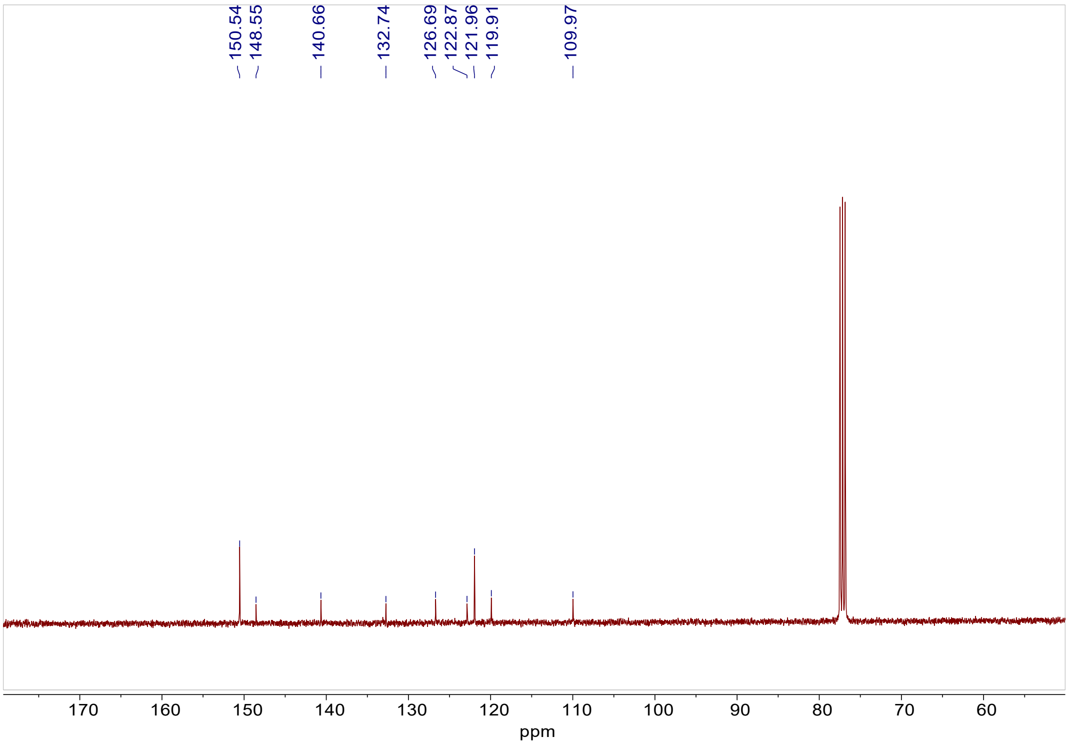

Fig. S4. ^{13}C NMR (100 MHz) spectrum of monomer **3,6-TPy-BCz** recorded in $CDCl_3$ at RT.

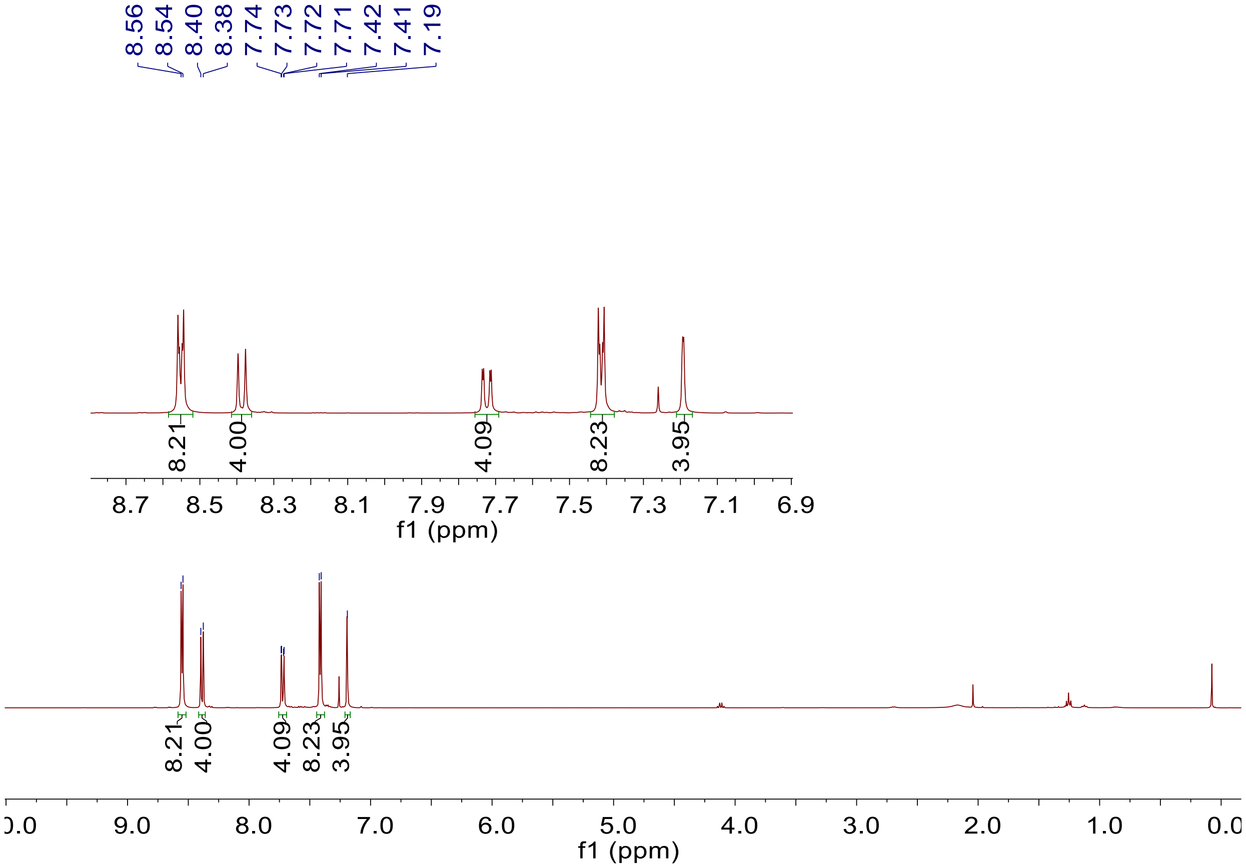

Fig. S5. ^{1}H NMR (400 MHz) spectrum of monomer **2,7-TPy-BCz** recorded in $CDCl_3$ at RT.

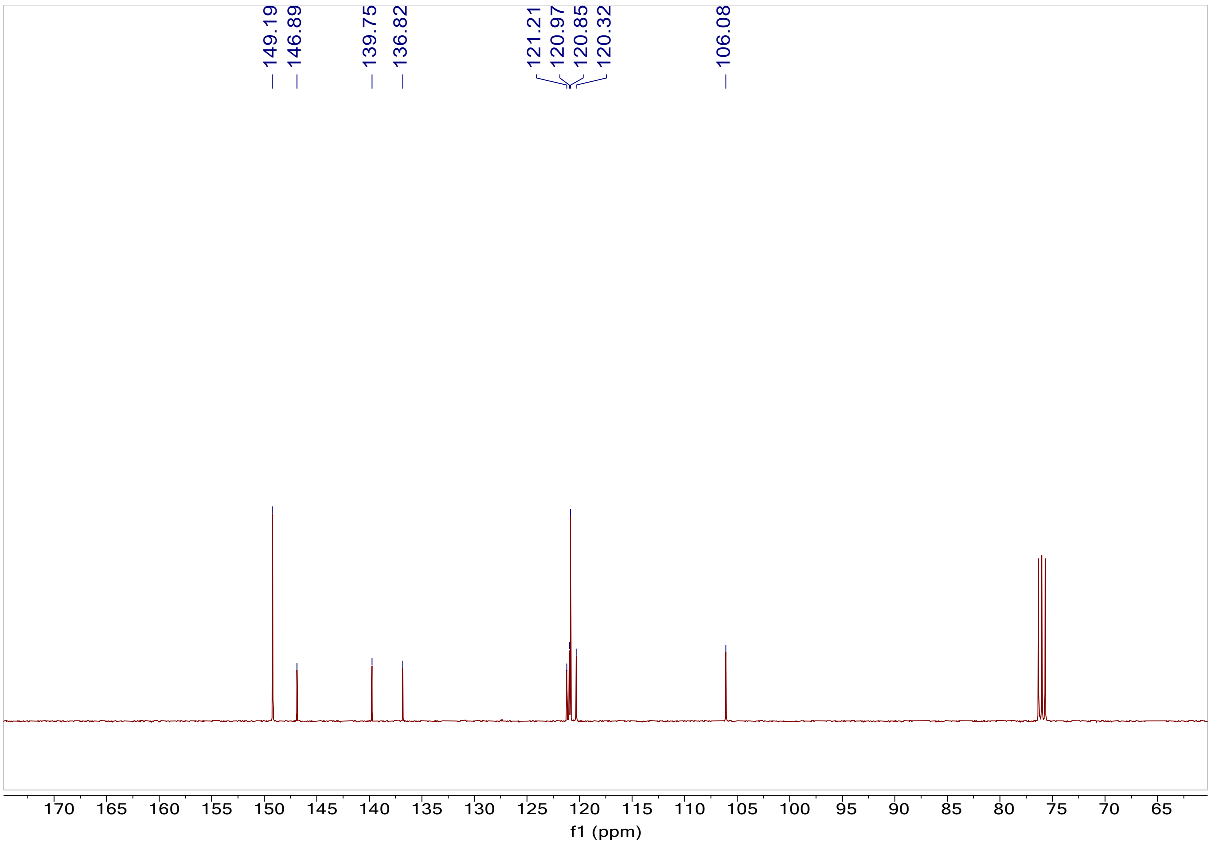

Fig. S6. ^{13}C NMR (100 MHz) spectrum of monomer **2,7-TPy-BCz** recorded in $CDCl_3$ at RT.

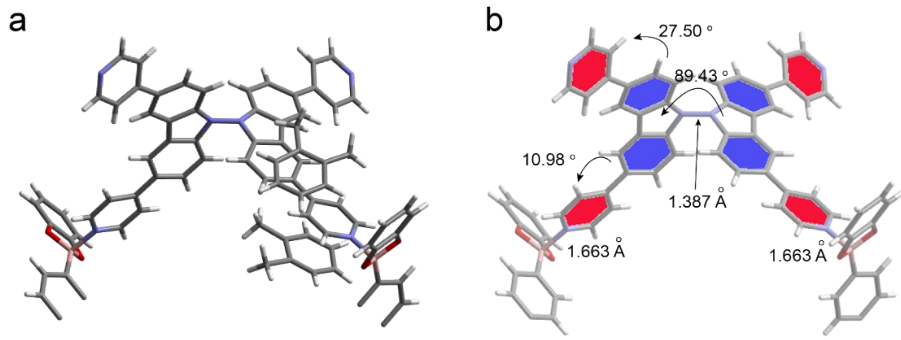

Fig. S7. (a) The asymmetric unit in **CityU-50** single crystal. (b) 3,6-TPy-BCz monomer and the two half BDBB moieties to which it is connected in the crystal structure.

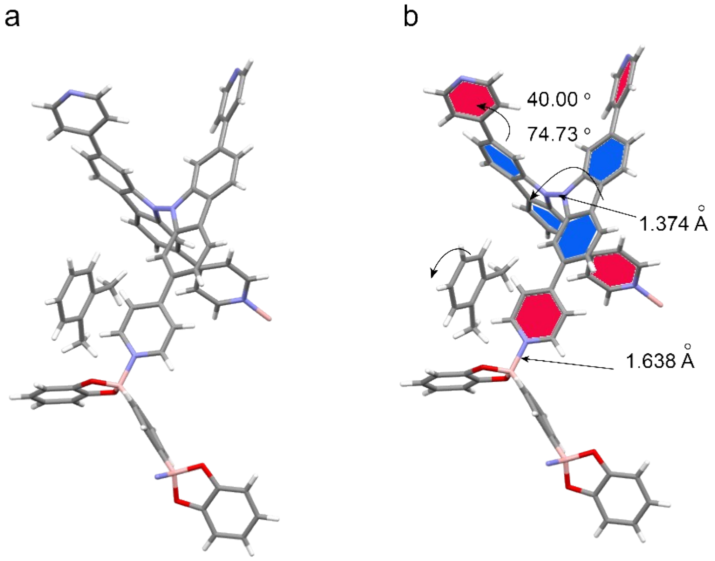

Fig. S8. (a) The asymmetric unit in **CityU-60** single crystal. (b) 2,7-TPy-BCz monomer and the two half BDBB moieties to which it is connected in the crystal structure.

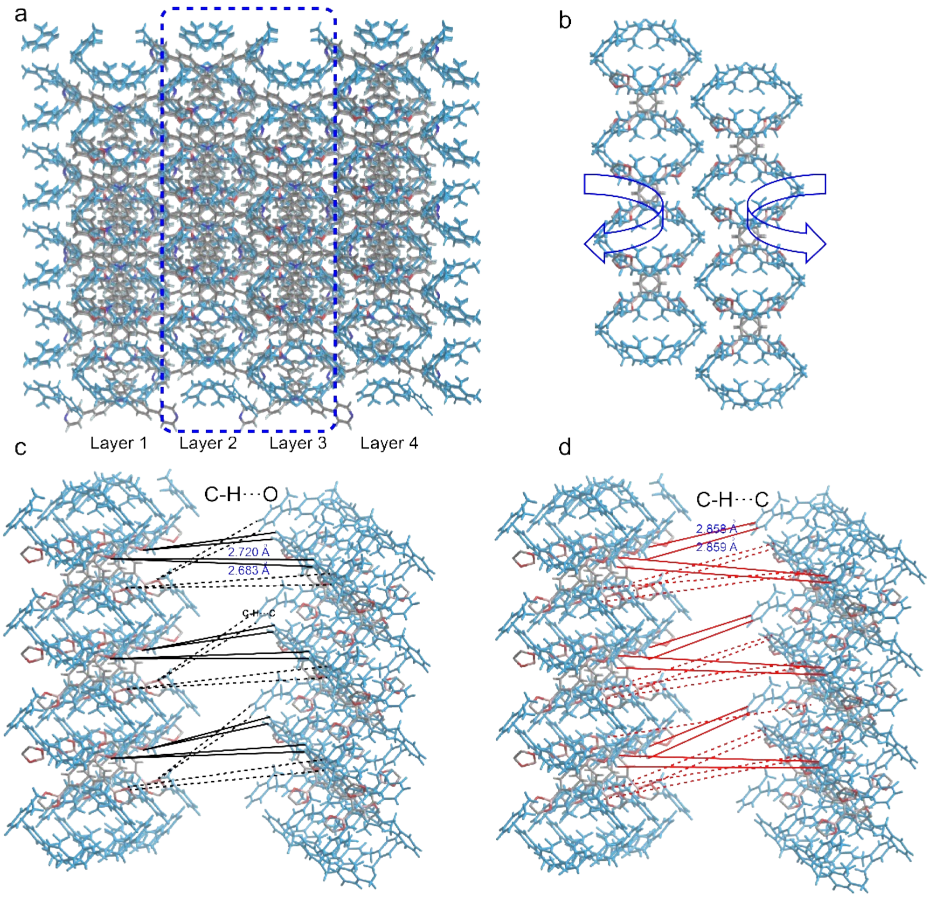

Fig. S9. Simplified illustration of the interactions between the solvent molecules and the polymer chains in **CityU-50.**

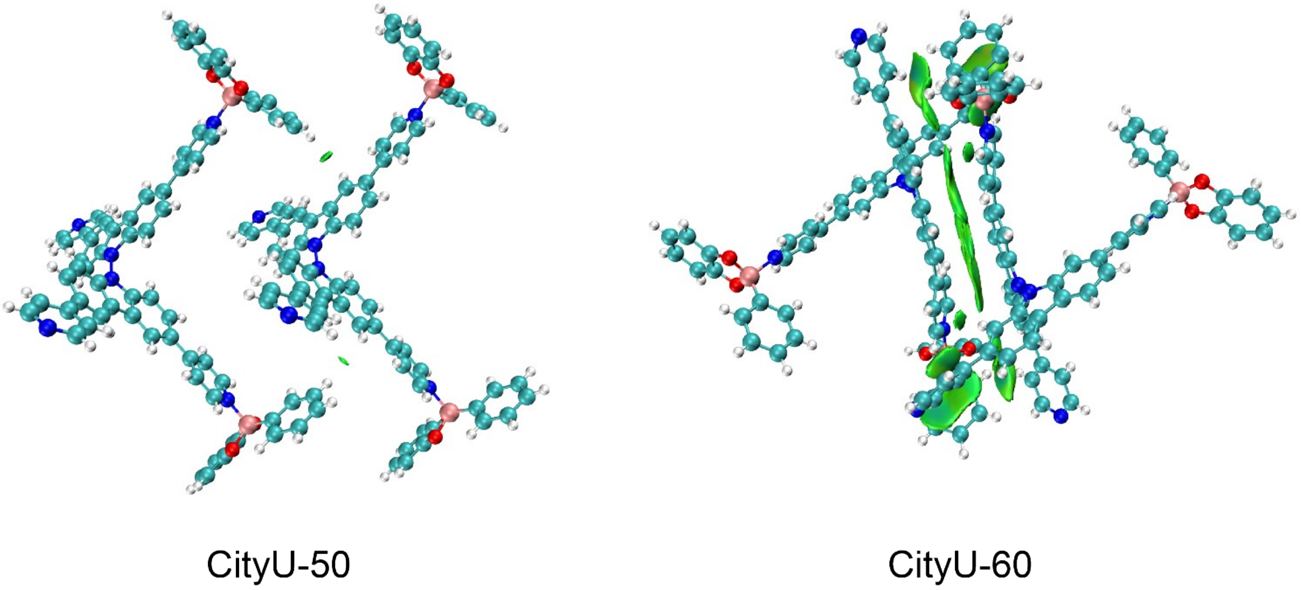

Fig. S10. The local 3D π-π interactions plot of dimer fragments without solvent molecule in **CityU-50** and **CityU-60** with an iso-surface value of 0.5.

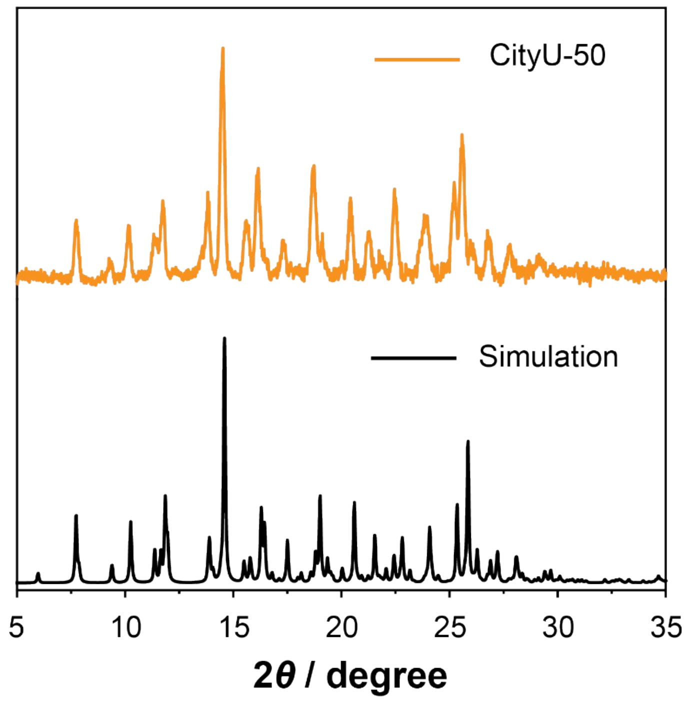

Fig. S11. Experimental and simulated Powder X-ray diffraction patterns of **CityU-50**.

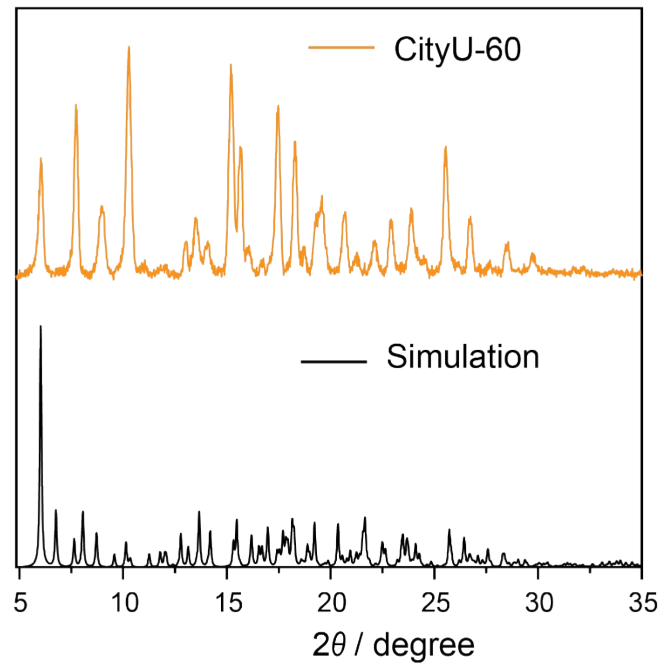

Fig. S12. Experimental and simulated Powder X-ray diffraction patterns of **CityU-60**.

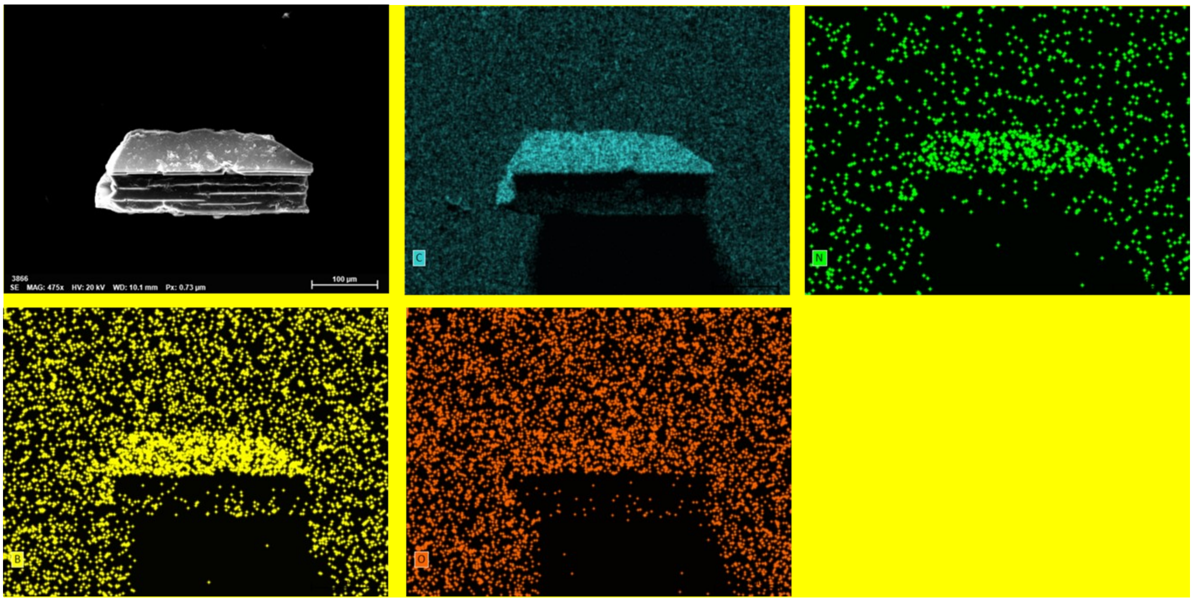

Fig. S13. SEM images and the corresponding EDS element mappings of **CityU-50**.

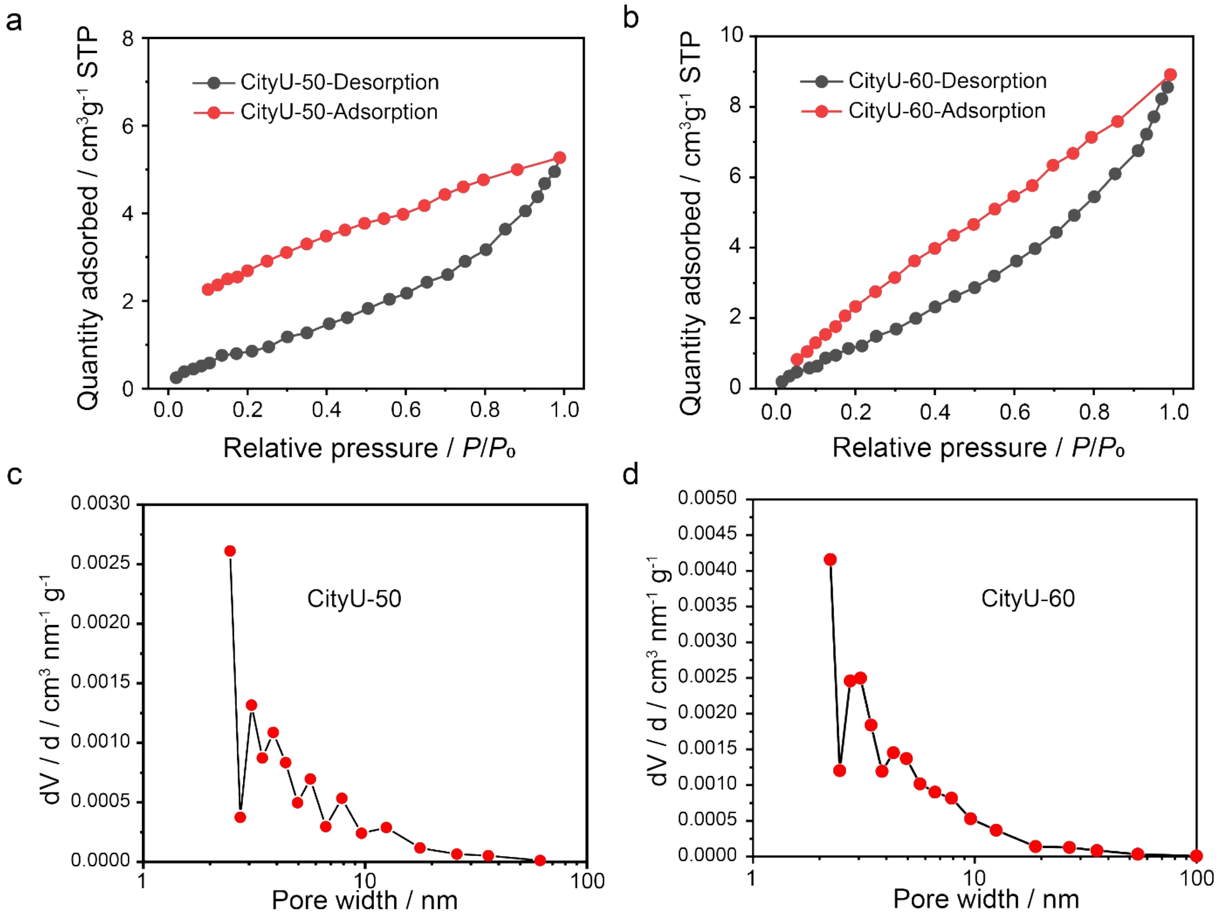

Fig. S14. N_2 isotherms of **CityU-50** and **CityU-60** at 77 K and the pore width distributions based on Barrett–Joyner–Halenda (BJH) adsorption mode.

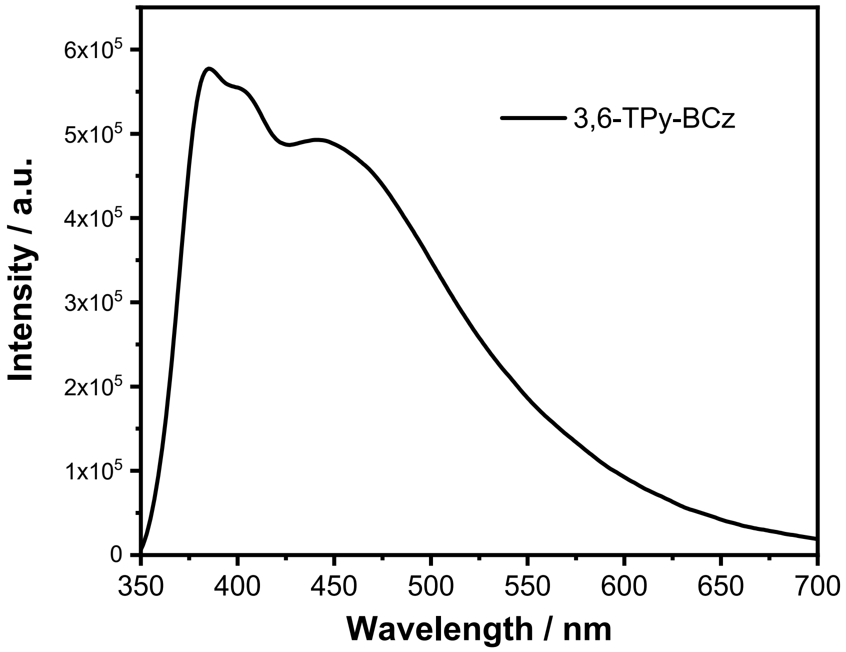

Fig. S15. The photoluminescence spectrum of the 3,6-TPy-BCz monomer in powder.

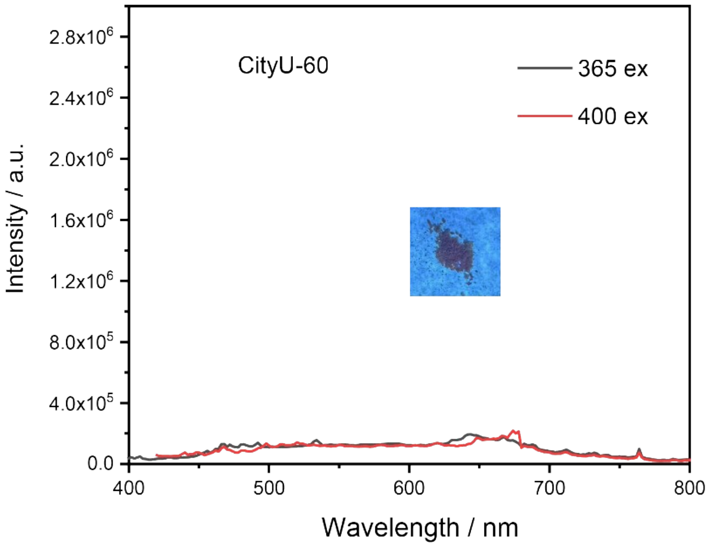

Fig. S16. The photoluminescence spectra of the **CityU-60** under different excitation wavelengths.

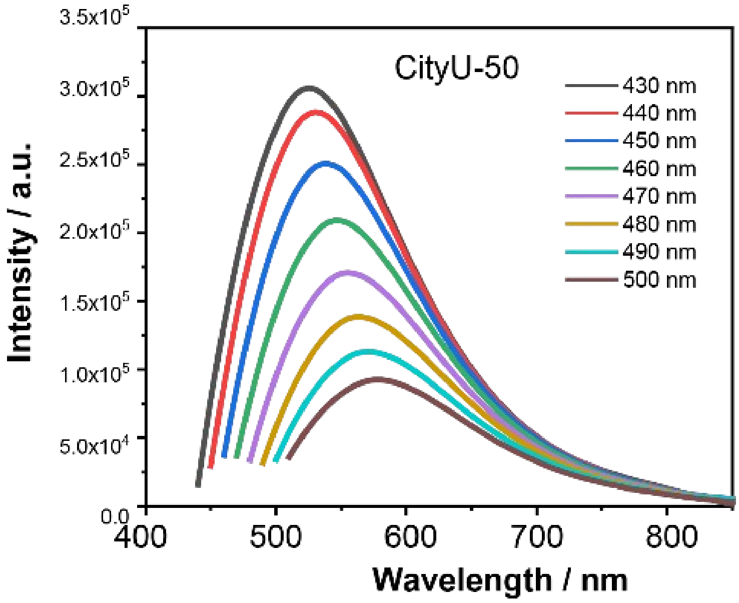

Fig. S17. The photoluminescence spectra of the **CityU-50** under different excitation wavelengths.

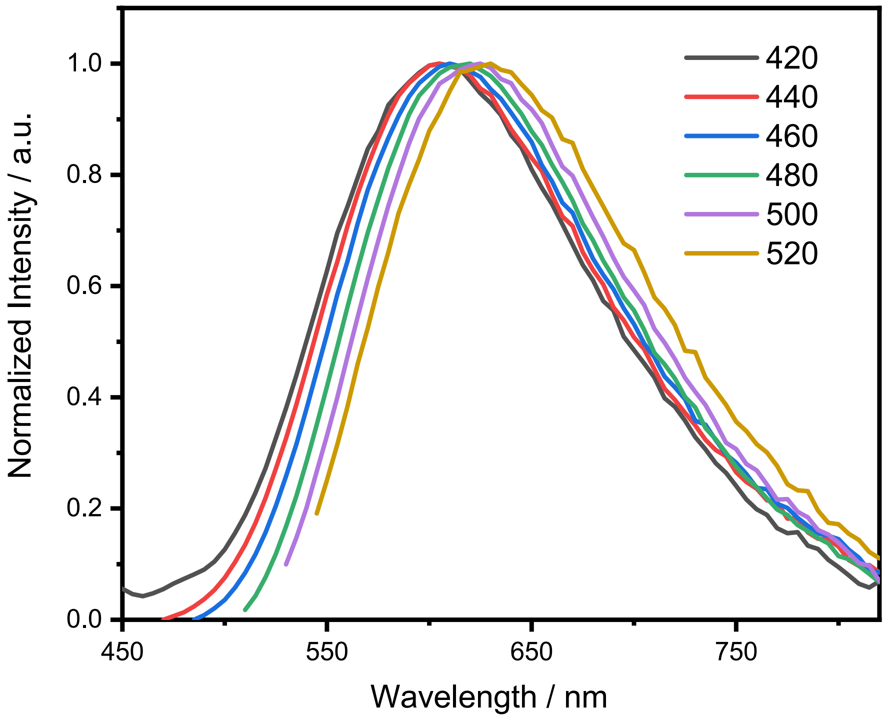

Fig. S18. Excitation-dependent photoluminescence of **CityU-50-1**, recorded under ambient conditions at 298 K.

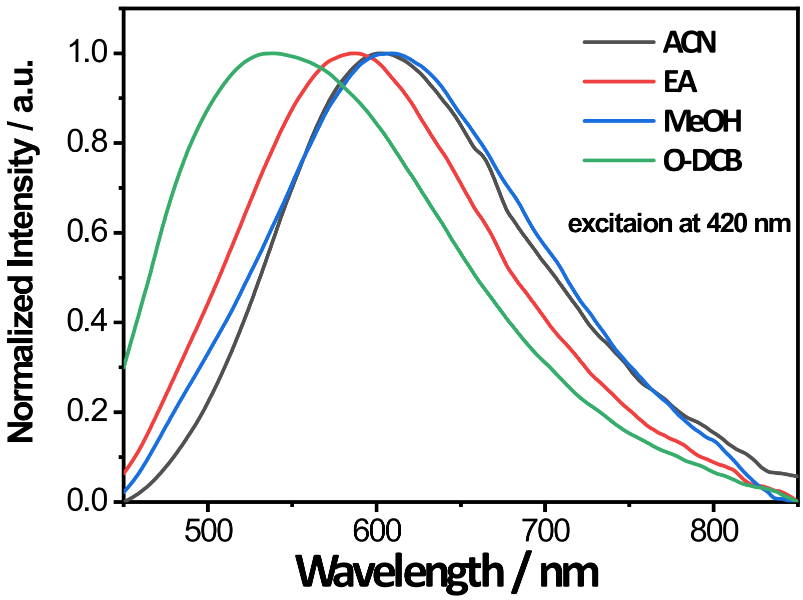

Fig. S19. The luminescence spectra of **CityU-50** treated with various solvents under 420 nm excitation.

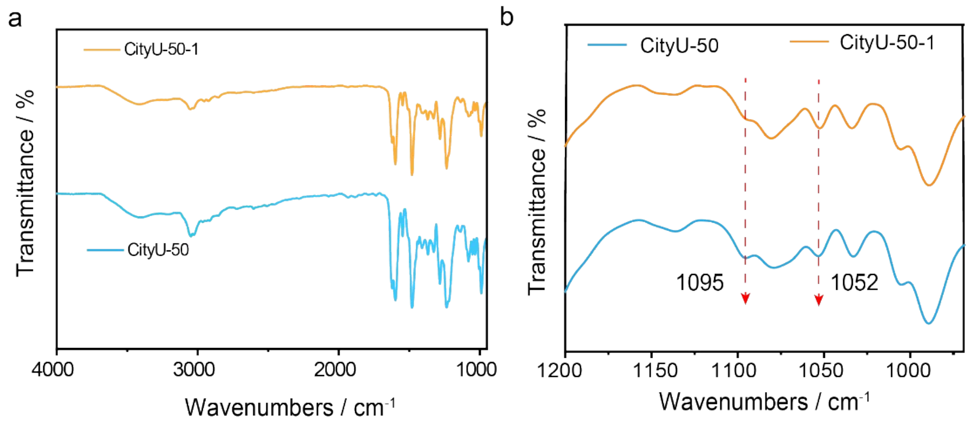

Fig. S20. FT-IR spectra of **CityU-50** and **CityU-50-1 (CityU-50** after treated with n-hexane).

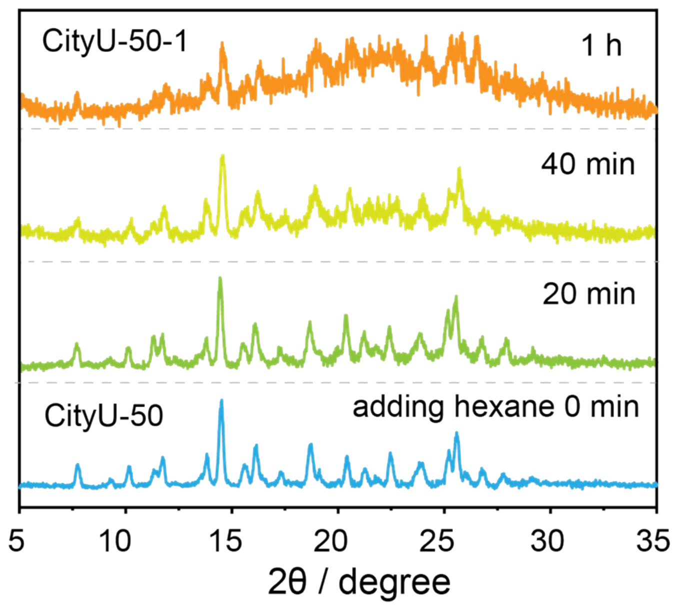

Fig. S21. The powder X-ray diffraction (PXRD) patterns of **CityU-50** samples treated with *n*-hexane at different soak time.

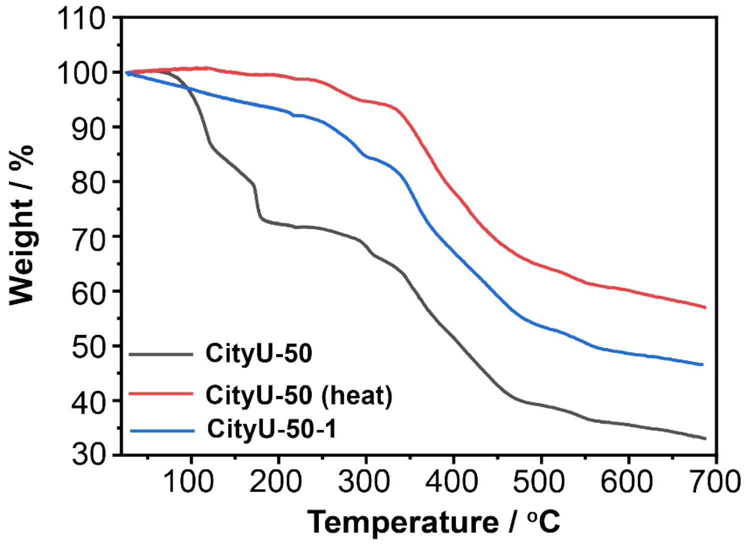

Fig. S22. Thermogravimetric analysis (TGA) spectra of **CityU-50** before and after heating at 150 °C and hexane immersion for 24 h. The weight loss of **CityU-50** before 300 °C can be attributed to the departure of solvent molecules (o-xylene).

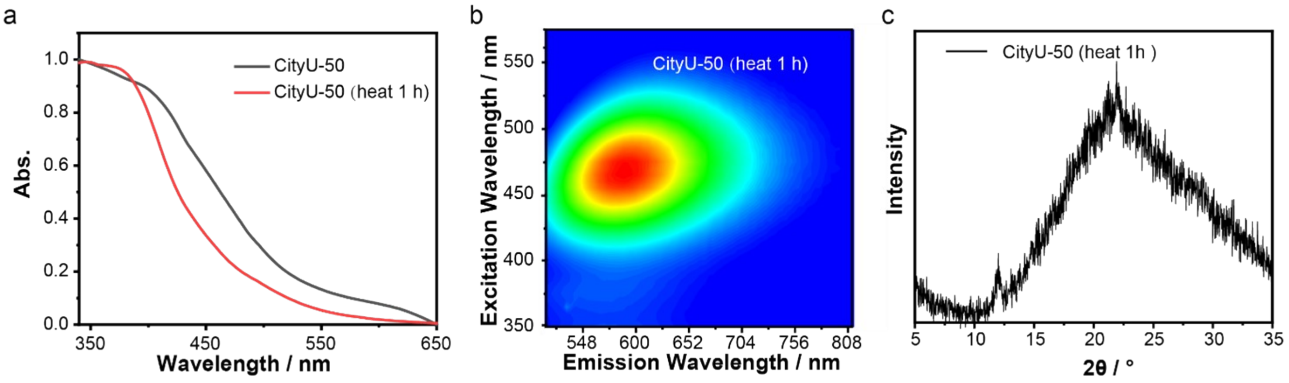

Fig. S23. (a) The absorption, (b) PXRD, and (c) photoluminescence spectrum of **CityU-50** after heating for 1 h at 150 °C.

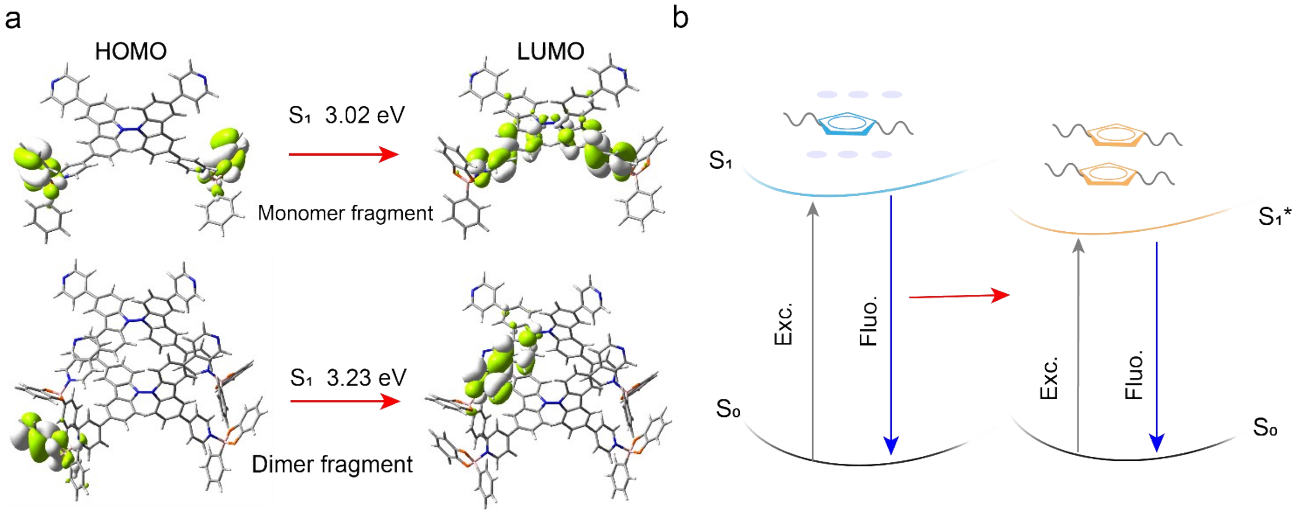

Fig. S22. (a) Illustrations showcasing electron cloud distributions of monomer and dimer fragment at ground state. (b) Proposed mechanism for tunable emission of **CityU-50**. The highest occupied molecular orbitals (HOMOs) of molecule fragment are primarily localized on the B-aryl rings, while the lowest unoccupied molecular orbitals (LUMOs) are mainly localized on the carbazole planes. This distinctive charge transfer characteristic facilitates the generation of photoluminescence of CityU-50. In contrast, for dimer fragments, the LUMOs are distributed across the carbazole planes in another molecules. Notably, dimer fragments exhibit a lower energy level in the excited singlet state relative to the monomer. These findings suggest that aggregation enhances the intermolecular charge transfer, leading to a redshift in both luminescence and absorption.

Table S1. Crystallographic data for single crystals of **CityU-50.**

sample	**CityU-50**<br>CCDC:2414993
Empirical formula	$C_{47}H_{40}BN_3O_2$
Formula weight	689.63
Temperature/K	169.99(10)
Crystal system	monoclinic
Space group	C2/c
a/Å	24.5308(4)
b/Å	10.3508(2)
c/Å	31.7355(6)
α/°	90
β/°	111.309(2)
γ/°	90
Volume/Å^3	7507.2(3)
Z	8
ρ_{calc}g/cm^3	1.220
μ/mm^{-1}	0.578
F(000)	2912.0
Crystal size/mm^3	0.16 × 0.14 × 0.12
Radiation	Cu Kα (λ = 1.54184)
2Θ range for data collection/°	5.978 to 133.192
Index ranges	-29 ≤ h ≤ 29, -12 ≤ k ≤ 11, -37 ≤ l ≤ 30
Reflections collected	13690
Independent reflections	6646 [R_{int} = 0.0240, R_{sigma} = 0.0286]
Data/restraints/parameters	6646/18/557
Goodness-of-fit on F^2	1.018
Final R indexes [I>=2σ (I)]	R_1 = 0.0669, wR_2 = 0.1907
Final R indexes [all data]	R_1 = 0.0717, wR_2 = 0.1975
Largest diff. peak/hole / e Å^{-3}	0.51/-0.52

Table S2. Crystallographic data for single crystals of **CityU-60.**

sample	**CityU-60**<br>CCDC:2465520
Empirical formula	$C_{70}H_{50}B_2N_6O_4$
Formula weight	1060.78
Temperature/K	170.06 (10)
Crystal system	monoclinic
Space group	$P2_1/n$
a/Å	16.4184(2)
b/Å	17.0819(2)
c/Å	21.1989(2)
α/°	90
β/°	106.929(10)
γ/°	90
Volume/Å^3	5687.75(11)
Z	4
ρ_{calc}g/cm^3	1.239
μ/mm^{-1}	0.609
F(000)	2216.0
Crystal size/mm^3	0.13 × 0.12 × 0.1
Radiation	Cu Kα (λ = 1.54184)
2Θ range for data collection/°	7.646 to 133.202
Index ranges	-19 ≤ h ≤ 19, -18 ≤ k ≤ 20, -25 ≤ l ≤ 25
Reflections collected	47329
Independent reflections	10041 [R_{int} = 0.0234, R_{sigma} = 0.0203]
Data/restraints/parameters	10041/1142/823
Goodness-of-fit on F^2	1.045
Final R indexes [I>=2σ (I)]	R_1 = 0.0465, wR_2 = 0.1229
Final R indexes [all data]	R_1 = 0.0553, wR_2 = 0.1295
Largest diff. peak/hole / e Å^{-3}	0.65/-0.39
